# Formation of surface states on Pb(111) by Au adsorption

**DOI:** 10.1038/s41598-023-28106-0

**Published:** 2023-01-30

**Authors:** Wei-Chuan Chen, Chin-Hsuan Chen, Angus Huang, Kaweng Lei, David Mikolas, Ming-kwan Dai, Je-Ming Kuo, Dai-Shien Lin, Cheng-Maw Cheng, H.-T. Jeng, S.-J. Tang

**Affiliations:** 1 Department of Physics, National Tsing Hua University, Hsinchu, 30013 Taiwan; 2 National Synchrotron Radiation Research Center (NSRRC), Hsinchu, 30076 Taiwan; 3 Institute of Physics, Academia Sinica, Taipei, 11529 Taiwan; 4 Physics Division, National Center for Theoretical Sciences, Taipei, 10617 Taiwan

**Keywords:** Surfaces, interfaces and thin films, Surfaces, interfaces and thin films, Electronic structure

## Abstract

Using low-energy electron diffraction and angle-resolved photoemission spectroscopy, we investigated the lattice and electronic structures of the Pb(111) surface upon the adsorption of Au atoms at the low temperature T = 40 K. Unlike earlier results showing the formation of PbAu-alloy layers at room temperature, we found that Au atoms form a ultra-thin superstructure, Au/Pb(111)-3 × 3, on top of the Pb(111) surface. Moreover, three surface-state bands, S_1_, S_2_, and S_3_, are induced within and immediately adjacent to the Pb bulk projected band gap centered at the surface zone boundary ${\overline{\text{M}}}_{Pb(111)}$ at the energies of − 0.02, − 1.05, and − 2.56 eV, respectively. First-principles calculation based on Au/Pb(111)-3 × 3 confirms the measured surface-state bands among which the most interesting are the S_1_ and S_3_ surface states. They are derived from surface resonances in Pb(111). Moreover, S_1_, which disperses across Fermi level, exhibits a large anisotropic Rashba splitting with α of 1.0 and 3.54 eVÅ in the two symmetry directions centered at ${\overline{\text{M}}}_{Pb(111)}$. The corresponding Rashba splitting of S_1_ bands in Cu/Pb(111)-3 × 3 and Ag/Pb(111)-3 × 3 was calculated for comparison.

## Introduction

Bulk Pb is well known to be a conventional *s*-wave superconductor with a fairly high transition temperature of superconductivity *T*_*C*_ of 7.23 K^1^. Based on the measured cyclotron effective mass, the renormalization factor λ (the so-called electron–phonon coupling strength) was extracted to have a high value up to 1.5^2^. It was not until 1980s that attention started to focus on the atomically uniform Pb thin films, for which measured *T*_*C*_ values exhibit bilayer-oscillatory dependence on thickness^3^. The same oscillatory behavior was also observed for electron–phonon coupling strength λ by investigating the temperature dependence of hole lifetime of quantum well states (QWS)^4^. The bilayer oscillations of *T*_*c*_ and λ were attributed to the variation of density of states (DOS) at the Fermi level, exclusively determined by the energy positions of QWS. However, the superconductivity as well as the electron–phonon interaction at the surfaces of bulk Pb and Pb thin films have been long overlooked because of the lack of localized surface states (SS)^5,6^. On the other hand, the Pb atom itself has strong spin–orbit coupling (SOC)^7^; therefore a large Rashba effect at the surfaces of Pb bulk or thin films was expected but has never been observed for the same reason. Nevertheless, a weak Rashba effect was observed from QWS of flat Pb thin films^8^. This can be understood in light of the fact that the QWS wavefunction distributes between two interfaces (vacuum/film and film/substrate) and the out-of-plane potential gradients derived from both oppose. The Rashba effect is thus smeared out.

Therefore, to explore the superconductivity and Rashba effect at the Pb(111) surface, pronounced and localized SS’s are indispensable. The experimental observation of SS in metal surfaces was more difficult than in semiconductor surfaces^9^. The main reason is that metal crystals have partial bulk band gaps rather than the complete bulk band gaps that exist in semiconductors. The first experimental observation and proof of SS in a metal surface was made from the transition metal, W(100), by E. W. Plummer and J. W. Gadzuk^10^ using field emission measurement. Wurde et al.^5^ pointed out that the well separated *s*, *p*, and *d* bands in bulk Pb makes the formation of SS due to hybridization less possible than in transition metals. In addition, the higher coordination of Pb atoms at nearest and next-nearest neighbors on the (111) surface compared to (100) and (110) surfaces makes SS even less pronounced at the (111) surface^5^. Induction of surface localized electronic structures by deposition of few foreign atoms to form superstructures was mostly on semiconductor surfaces^11–14^, but less frequently on metals surfaces, e.g. Pb/Cu(100)-c(4 × 4)^15^, Bi/Au(110)-1 × 4^16^, and Pb/Au(111)-(5.77 × 5.77) R21.5^17^. Such a method has been rarely applied to Pb(111) because of its interactive nature to form alloy surfaces with foreign atoms^18–23^. In this paper, we deposited Au atoms onto Pb(111) surface specifically at a low temperature of 40 K and consequently a superstructure phase of Au/Pb(111)-3 × 3 formed. Moreover, three new SS bands were observed to exist within and around the Pb bulk projected band gap centered at the surface zone boundary ${\overline{\text{M}}}_{Pb(111)}$. Their characteristics as well as their contributions towards the interesting properties at the surfaces are discussed.

## Results and discussion

**Figure 1:**
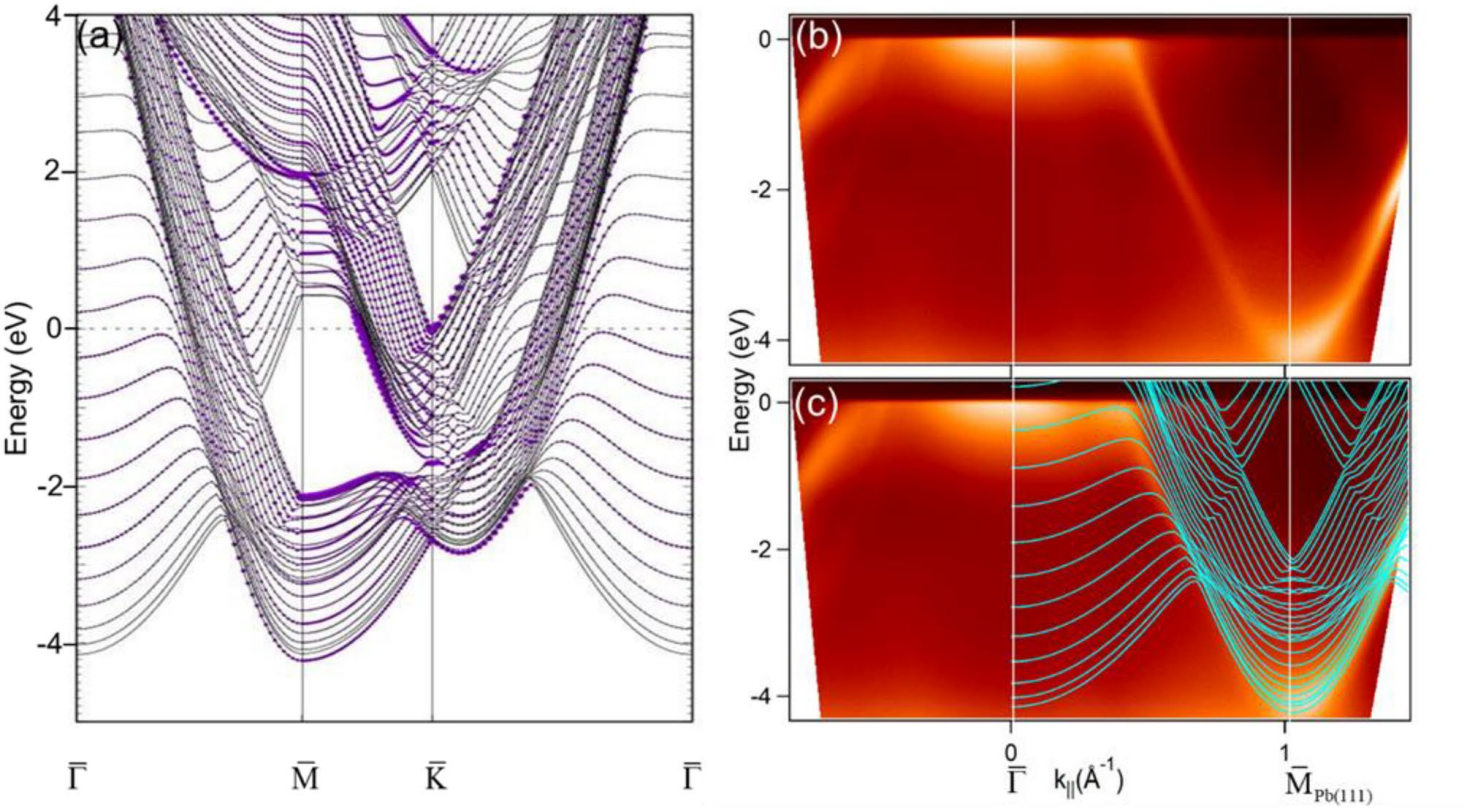
(**a**) The calculated energy band structures of a 24-layer Pb(111) slab. The purple color indicates the weight of the surface component. (**b**) The measured energy band structure along the symmetry direction $\overline{\Gamma }{\overline{\text{M}}}_{{{\text{Pb}}(111)}}$ at RT. (**c**) Overlap of the calculated bands in (**a**) with the measured ones in (**b**).

Figure 1a shows the calculated energy band structures of a 24-layer Pb(111) slab. The purple color indicates the weight of the surface component. As reported previously^5^, there is a non-inverted 6*sp* bulk band gap ranging from − 8.3 to − 3.7 eV projected to the surface zone center $\overline{\Gamma }$. With the top band edge *p*-type and bottom band edge *s*-type, the SS is unlikely to exist within the band gap according to Shockley’s model^9^. Our focus is rather at the surface zone boundary ${\overline{\text{M}}}$, where a projected bulk band gap ranges between − 2.14 eV at the bottom bulk band edge (BBBE) and 0.42 eV at the top bulk band edge (TBBE). This gap originates from the strong SOC of 6*p* bands^5^; the main orbital components of TBBE and BBBE are both *p*-type, which doesn’t fit Shockley’s model, either^9^. Figure 1b shows the measured energy band dispersions of Pb(111) at RT in the symmetry direction $\overline{\Gamma }{\overline{\text{M}}}_{Pb(111)}$(${\overline{\text{M}}}_{{{\text{Pb}}(111)}}$ at 1.03 Å^−1^). This band gap spans in k space as a rhombus centered at ${\overline{\text{M}}}_{Pb(111)}$ due to the mirror-image symmetry with respect to it. The calculated subbands in Fig. 1a are superimposed onto the measured one as shown in Fig. 1c. Note that the calculation is based on a slab model so the calculated subbands are mainly discrete QWS bands. However, both the bulk band edges of the rhombus gap centered at ${\overline{\text{M}}}_{Pb(111)}$ as well as the measured Pb bulk band dispersing from the energy just below Fermi level at $\overline{\Gamma }$ down to about − 4 eV at ${\overline{\text{M}}}_{Pb(111)}$ are well reproduced. In their calculation for Pb(111) electronic structures, Wurde et al.^5^ predicted that there are two surface resonance (SR) states near BBBE and TBBE at ${\overline{\text{M}}}_{Pb(111)}$ [S_1_ and S_2_ of Fig. 3 in Ref. 5]. However, no measured counterparts have been ever observed.

**Figure 2:**
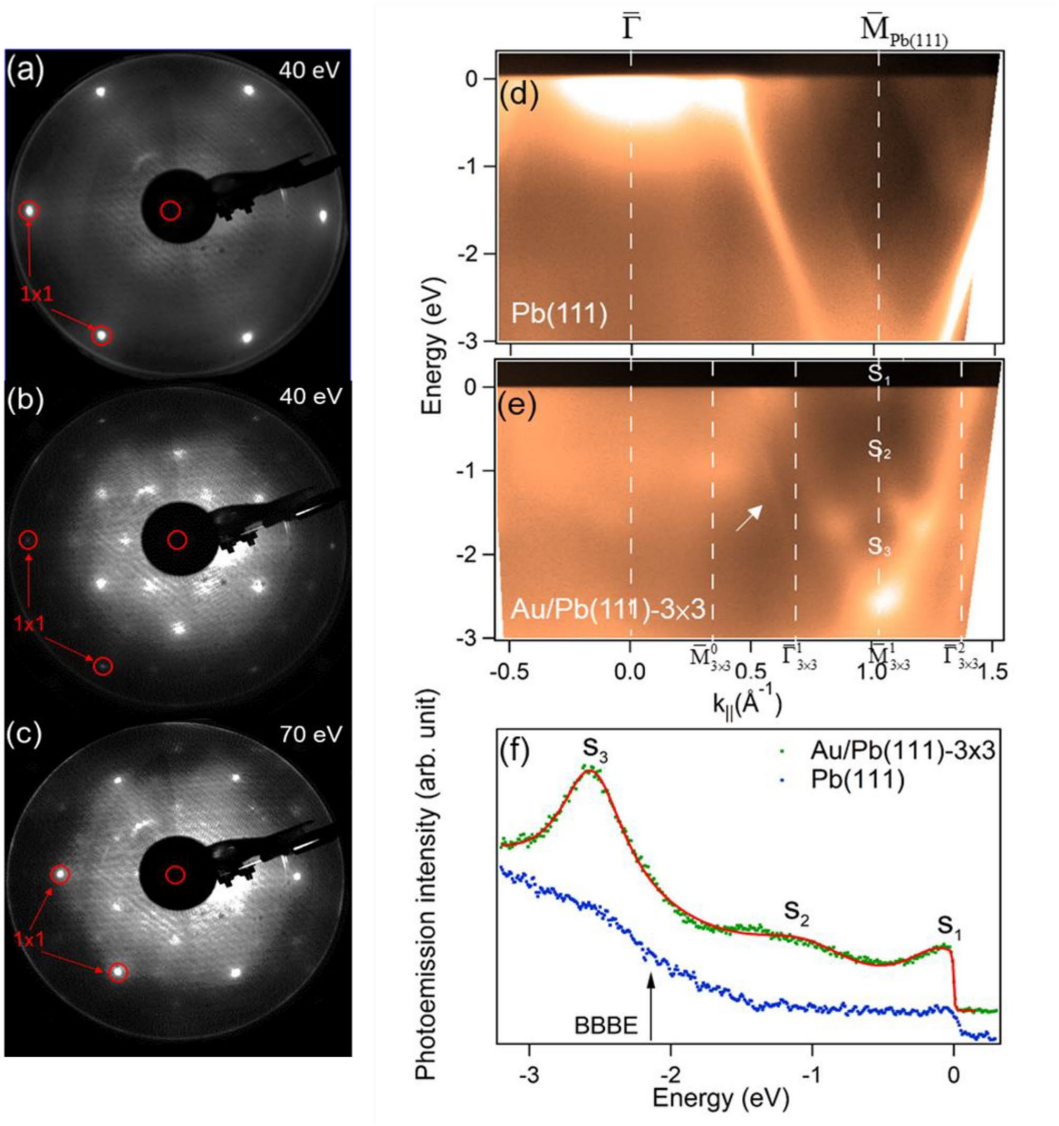
(**a**) LEED pattern of a clean Pb(111) surface. (**b**) and (**c**) LEED patterns of Pb(111) adsorbed with dilute Au atoms at electron energies of 40 eV and 70 eV. (**d**) The measured energy band structures of Pb(111) along the symmetry direction $\overline{\Gamma }{\overline{\text{M}}}_{{{\text{Pb}}(111)}}$. (**e**) The measured energy band structures of Au/Pb(111)-3 × 3 along the symmetry direction $\overline{\Gamma }{\overline{\text{M}}}_{{{\text{Pb}}(111)}}$($\overline{\Gamma }_{3 \times 3}^{{0}} - {\overline{\text{M}}}_{3 \times 3}^{{0}} - \overline{\Gamma }_{3 \times 3}^{1} - {\overline{\text{M}}}_{3 \times 3}^{1} - \overline{\Gamma }_{3 \times 3}^{2}$). (**f**) The extracted EDCs at $\overline{\mathrm{M} }$_Pb(111)_ of clean Pb(111) (blue) and Au/Pb(111)-3 × 3 (green). The red curve is the fitting to the EDC of the Au/Pb(111)-3 × 3.

We cooled down the Pb(111) substrate to 40 K and then deposited few Au atoms onto it. Figure 2a,b exhibit LEED patterns of Pb(111) before and after Au deposition at an electron energy of 40 eV. The red circles enclose the Pb(111)-1 × 1 spots and other spots emerging between them after Au deposition are 3 × 3 spots. Note that the 2/3 order spots of 3 × 3 are coincident with $\sqrt 3 \times \sqrt 3 {\text{R30}}^\circ$ spots; however, the intensities of 1/3 and 2/3 order spots, as seen in Fig. 2b, are about even, making the possibility of the existence of $\sqrt 3 \times \sqrt 3 {\text{R30}}^\circ$ phase unlikely. Figure 2c also shows the LEED pattern after Au deposition at 70 eV, where the Pb(111)-1 × 1 spots are brighter than those in Fig. 2b due to the change in diffraction conditions. The measured energy band structures of Pb(111) and Au/Pb(111)-3 × 3 are displayed in Fig. 2d,e, respectively for comparison. As observed, the rhombus bulk band gap centered at ${\overline{\text{M}}}_{{\text{Pb(111)}}}$ is occupied by two SS bands, S_1_ and S_2_. A third band, S_3_, disperses beneath the BBBE of the rhombus bulk band gap. Photon-energy dependent measurement, as shown in the Fig. S1 of supplementary material, confirms their 2-dimensional (2D) nature. The faint trace of the measured intense Pb bulk band in Fig. 2d can be still detected, as indicated by an arrow, in the measured spectrum of Au/Pb(111)-3 × 3 in Fig. 2e. When the superstructure Au/Pb(111)-3 × 3 forms, the 3 × 3 lattice can inflict an extra reciprocal vector $\vec{G}_{3 \times 3}$ to the momentum of photoelectrons**.** This means that the final-state band is altered and the observed bulk band observed in Pb(111) due to the direct transition can diminish^24^. The energy distribution curves (EDCs) of Pb(111) and Au/Pb(111)-3 × 3 at ${\overline{\text{M}}}_{Pb(111)}$ (${\overline{\text{M}}}_{3x3}^{1}$) are extracted and shown in Fig. 2f. The peaks for S_1_ , S_2_, and S_3_ are at the energy positions of − 0.02, − 1.05, and − 2.56 eV based on a fit of three Lorentzians plus a polynomial background, multiplied by a Fermi function. It is noteworthy that S_3_ is closest to the energy position of BBBE so it should be related to the SR at ${\overline{\text{M}}}_{Pb(111)}$ in Pb(111). The arrow indicates the energy position of BBBE projected to Pb(111).

**Figure 3:**
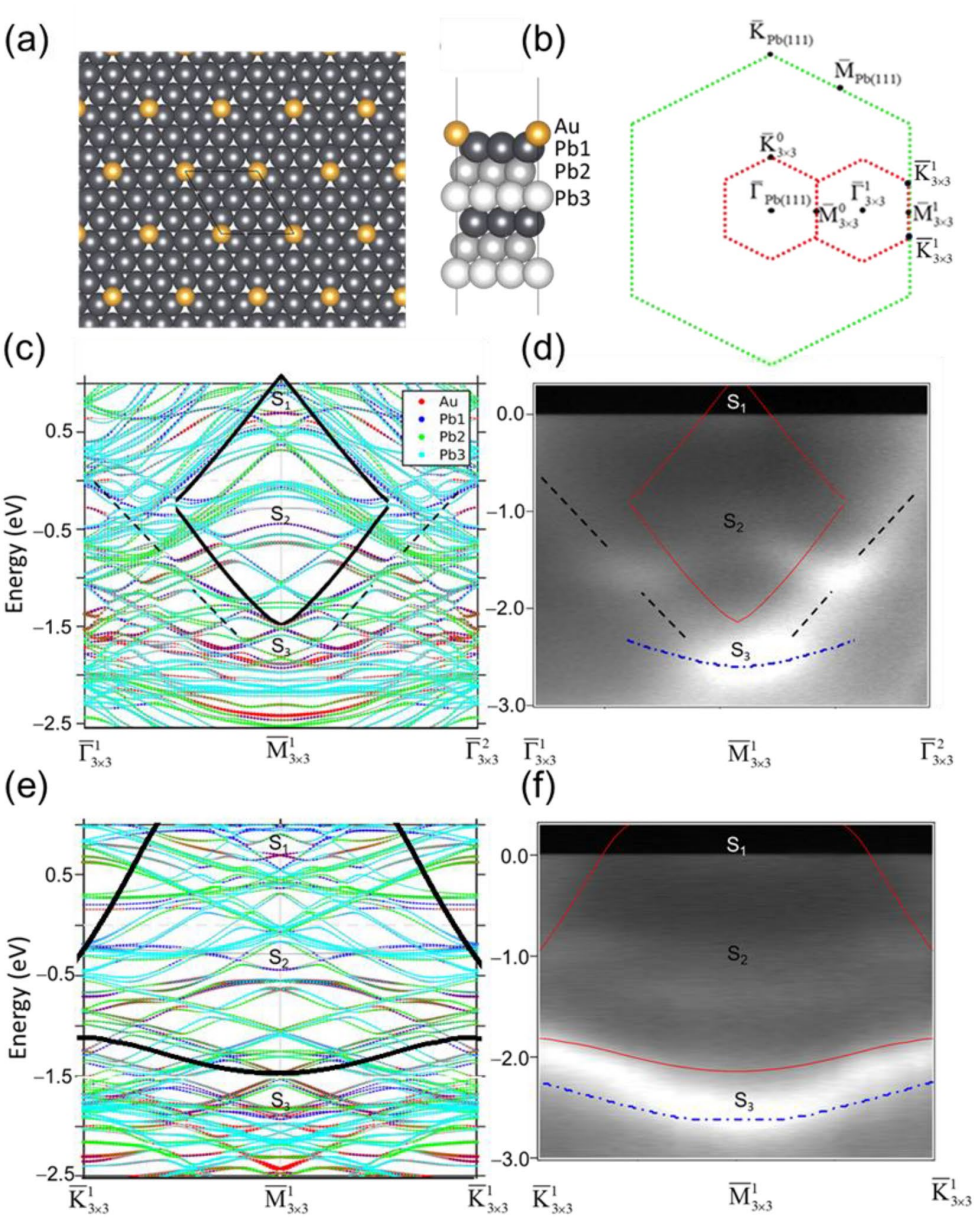
(**a**) The resulting Au/Pb(111)-3 × 3 lattice structure deduced from DFT calculation in top view and side view. (**b**) The surface brouillon zones of Pb(111) and Au/Pb(111)-3 × 3. (**c**) and (**d**) The calculated and measured energy band dispersions of Au/Pb(111)-3 × 3 in the regime $\overline{\Gamma }_{3 \times 3}^{1} - {\overline{\text{M}}}_{3 \times 3}^{1} - \overline{\Gamma }_{3 \times 3}^{2}$. (**e**) and (**f**) The calculated and measured energy band dispersions of Au/Pb(111)-3 × 3 in the regime ${\overline{\text{K}}}_{3 \times 3}^{1} - {\overline{\text{M}}}_{3 \times 3}^{1} - {\overline{\text{K}}}_{3 \times 3}^{1}$. The regions of Pb bulk projected band gap in both symmetry directions are enclosed by dark (red) frames in calculated (measured) bands.

First principles calculation was implemented using a Au/Pb(111)-3 × 3 superstructure on a 6-ML Pb slab. In the relaxed configuration the Au atoms sit at the (fcc) hollow sites of Pb(111) as shown in the top view of the slab model in Fig. 3a. The energies for Au atoms at other positions such as top site and bridge site are higher than at hollow sites according to the calculation (See Fig. S2 of supplementary material). The side view shows the fcc stacking of the top Au atom and the Pb atoms beneath, among which the Pb atoms in the top three layers are labeled. The resulting calculated bands within the two symmetry regimes $\overline{\Gamma }_{3 \times 3}^{1} - {\overline{\text{M}}}_{3 \times 3}^{1} - \overline{\Gamma }_{3 \times 3}^{2}$ and ${\overline{\text{K}}}_{3 \times 3}^{1} - {\overline{\text{M}}}_{3 \times 3}^{1} - {\overline{\text{K}}}_{3 \times 3}^{1}$ around the bulk band gap are shown in Fig. 3c, e. The surface brillouin zones of Pb(111) and Au/Pb(111)-3 × 3 are plotted in Fig. 3b. Figure 3d, f show the measured counterparts in the same regimes for a clear comparison. Note that an offset of 0.7 eV between the measurement and calculation is applied to achieve a reasonable match. The frames enclosing the projected bulk band gaps in both symmetry directions are superimposed. The sizes of the red, deep blue, green, and light blue circles represent the weights of the calculated bands in the top Au and the first, the second and the third Pb layer, respectively. In Figs. S3 and S4 of the supplementary material, the corresponding EDCs and the further 2nd derivative image processing of the measured energy band structures in Fig. 3d, f are displayed to help clarify S_1_, S_2_, and S_3_ bands.

As one can see, the measured S_1,_ S_2_ and S_3_ bands correspond to the calculated counterparts with dominant red and deep blue colors. S_1_ band disperses across the Fermi level within the bulk band gap. S_2_ band disperses through the bulk band edge to fall partially into the gap while S_3_ band completely falls below BBBE and concentrates at ${\overline{\text{M}}}_{3 \times 3}^{1} (\overline{\text{M}}_{{{\text{Pb}}(111)}} ).$ In the symmetry direction $\overline{\Gamma }_{3 \times 3}^{1} - {\overline{\text{M}}}_{3 \times 3}^{1} - \overline{\Gamma }_{3 \times 3}^{2}$, there is an extra upward band outside the gap, as indicated by the dark dashed lines, connecting from S_3_ to S_2_ and then extending toward Fermi level. It appears that this extra band corresponds to higher aggregation density of calculated QWS subbands of the slab model. The calculation (Fig. S5 of supplementary material) shows that those subbands have more or less Au components and hence can be considered as SRs. However, their surface weights are not as large as those of S_2_ and S_3_ so the intensity of the extra band is substantially lower than them in Fig. 3d. In the symmetry direction ${\overline{\text{K}}}_{3 \times 3}^{1} - {\overline{\text{M}}}_{3 \times 3}^{1} - {\overline{\text{K}}}_{3 \times 3}^{1}$, such an extra band is not observed. Because the entire S_3_ band dispersion is merely below the BBBE, much more extended dispersion of S_3_ band is observed in this direction. Although the calculated SS bands still extend out of the rhombus Pb bulk projected band gap, only those within and just outside the gap are observed in the measurement. Calculation also shows the measured S_3_ band is actually composed of aggregated SR subbands and S_2_ covers two bands, the lower one of which has more surface weight in the symmetry direction ${\overline{\text{K}}}_{3 \times 3}^{1} - {\overline{\text{M}}}_{3 \times 3}^{1} - {\overline{\text{K}}}_{3 \times 3}^{1}$ so it can be identified in the measured counterpart (Fig. 3f).

Charge of the S_2_ state at ${\text{M}}_{{{3} \times {3}}}^{{1}} ({\text{M}}_{{\text{Pb(111)}}} )$ distributes as 43.14, 25.49, 5.88, and 9.8% in the Au and first three Pb layers (Fig. S6 of supplementary material). Its large surface weight is in line with its energy position located in the middle of bulk projected band gap^9^. The main contributing Au and Pb orbitals are Au-6*s* and Pb-6*p*_*x*_-6*p*_*y*_. S_1_ state is pertinent to the conducting property of Au/Pb(111)-3 × 3 phase since it is near to and crosses the Fermi level. At ${\text{M}}_{{{3} \times {3}}}^{{1}} ({\text{M}}_{{\text{Pb(111)}}} )$, charge is distributed at 45.0%, 17.5%, 8.75%, and 6.25% in the Au layer and the top three Pb layers (Fig. S6 of supplementary material). The main contributing Au and Pb orbitals are Au-6*s* and Pb-6*p*_*x*_-6*p*_*y*_. Note that Wurde et al. predicted a SR band in Pb(111) at ${\overline{\text{M}}}_{{{\text{Pb(111}})}}$ right at TBBE of 0.42 eV [The band labeled “S_2_” of Fig. 3 of Ref. 5]. Therefore, it is likely that the S_1_ state of Au/Pb(111)-3 × 3 is derived from this SR although its energy descends to be below TBBE. The transition temperature, *T*_*C*_, of superconductivity is closely related to the DOS at the Fermi level, N(E_f_)^3^. Therefore, the S_2_ state can contribute to the surface superconductivity of Au/Pb(111)-3 × 3. The S_3_ band, with its energy position near BBBE, is likely derived from another SR of Pb(111) [The band labeled “S_1_” of Fig. 3 in Ref. 5] . The blue dashed-dot curves in Fig. 3d, f represent the calculated SR bands of Pb(111), extracted from Fig. 3 of Ref. 5 in two symmetry directions. It is evident that the SR band of Pb(111) resembles the S_3_ of Au/Pb(111)-3 × 3 except for a slight energy offset ~ 0.06 eV. S_3_ band charge at ${\text{M}}_{{{3} \times {3}}}^{{1}} ({\text{M}}_{{\text{Pb(111)}}} )$ distributes as 45.38, 12.61, 5.88, and 4.2% in the Au layer and the first three Pb layers (Fig. S6 of supplementary material). The main contributing Au and Pb orbitals are Au-5*d*_*xz*_-5*d*_*yz*_ and Pb-6*p*_*x*_-6*p*_*y*_. Compared to S_1_ and S_2_, S_3_ state has quite less charge in the top three Pb layers, indicating its longer decay length into the bulk.

**Figure 4:**
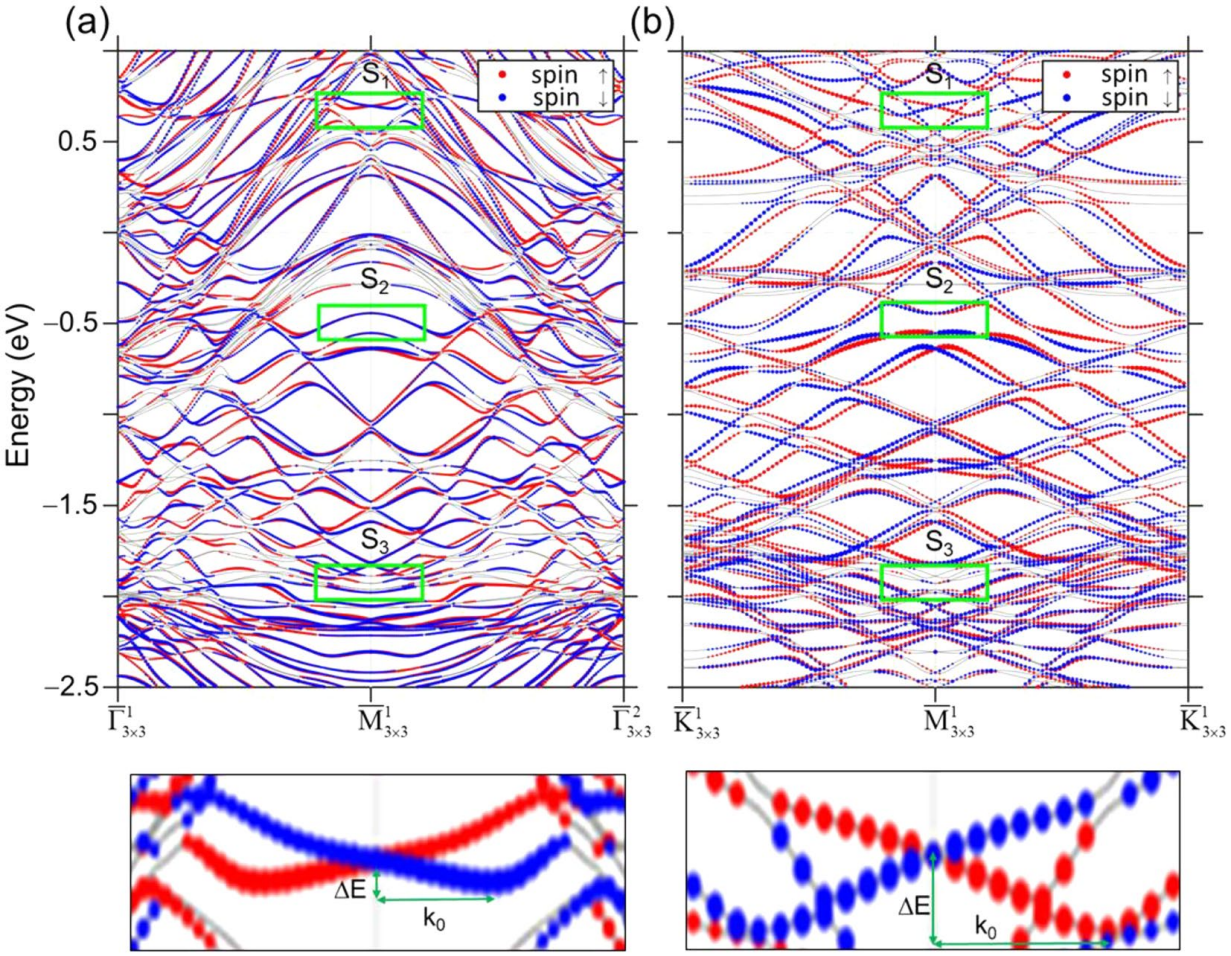
The calculated spin polarization of the Au/Pb(111)-3 × 3 energy band structures in the symmetry directions (**a**) $\overline{\Gamma }_{3 \times 3}^{1} - {\overline{\text{M}}}_{3 \times 3}^{1} - \overline{\Gamma }_{3 \times 3}^{2}$ and (**b**) ${\overline{\text{K}}}_{3 \times 3}^{1} - {\overline{\text{M}}}_{3 \times 3}^{1} - {\overline{\text{K}}}_{3 \times 3}^{1}$. Red and blue colors denote the in-plane spin polarizations in opposite directions. The insets below show the magnified views of S_1_ Rashba splitting.

The similarity of S_1_ and S_3_ bands of Au/Pb(111)-3 × 3 to the two SR bands at TBBE and BBBE, respectively, centered at ${\overline{\text{M}}}$ of Pb(111) points out that a SR state existing at the edge of a bulk band gap in the substrate that may at first seem irrelevant can turn much more pronounced after being covered with a superstructure or thin layer made of foreign atoms. Au atoms have a higher electronegativity than Pb (2.4 vs 1.9) so Au atoms attracts electron charges of SR in Pb(111) to cause more distribution of its wave function at the interface. Intriguingly, this begs the question “Does such SR wavefunction distribution enhance the Rashba effect that requires the breaking of inversion-symmetry through the interface?” Fig. 4a,b show the spin-polarization of the calculated subbands for Au/Pb(111)-3 × 3 in the symmetry directions, $\overline{\Gamma }_{3 \times 3}^{1} - {\overline{\text{M}}}_{3 \times 3}^{1} - \overline{\Gamma }_{3 \times 3}^{2}$ and ${\overline{\text{K}}}_{3 \times 3}^{1} - {\overline{\text{M}}}_{3 \times 3}^{1} - {\overline{\text{K}}}_{3 \times 3}^{1}$, respectively. Green rectangles enclose S_1_, S_2_, and S_3_ bands to guide the eye. As one can see, the Rashba splitting is negligible for S_2_, and S_3_ bands but relevant for S_1_. The extracted Rashba constant $\alpha { = }\frac{2\Delta E}{{k_{0} }}$ is 1.0 eVÅ (Δ*E* = 0.016 eV, *k*_0_ = 0.032 Å^−1^) in the symmetry direction $\overline{\Gamma }_{3 \times 3}^{1} - {\overline{\text{M}}}_{3 \times 3}^{1} - \overline{\Gamma }_{3 \times 3}^{2}$ and 3.54 eVÅ (Δ*E* = 0.0469 eV, *k*_0_ = 0.0275 Å^−1^) in ${\overline{\text{K}}}_{3 \times 3}^{1} - {\overline{\text{M}}}_{3 \times 3}^{1} - {\overline{\text{K}}}_{3 \times 3}^{1}$. The Rashba constants *α* of the SS of Au(111) and the QWS of Pb(111) thin films were measured to be 0.33^25,26^ and 0.044 eVÅ^8^, respectively. Therefore the absorbed Au 3 × 3 layer on Pb(111) not only promotes the original SR state but also significantly enhance Rashba effect of it. According to the charge-distribution percentage of S_1_, S_2_, and S_3_ over the top four layers, as described above, S_1_ has more charge than S_2_ at the top Au layer and the second Pb layer, hence triggering Rashba effect more effectively via considering the first Pb layer as the interface.

**Figure 5:**
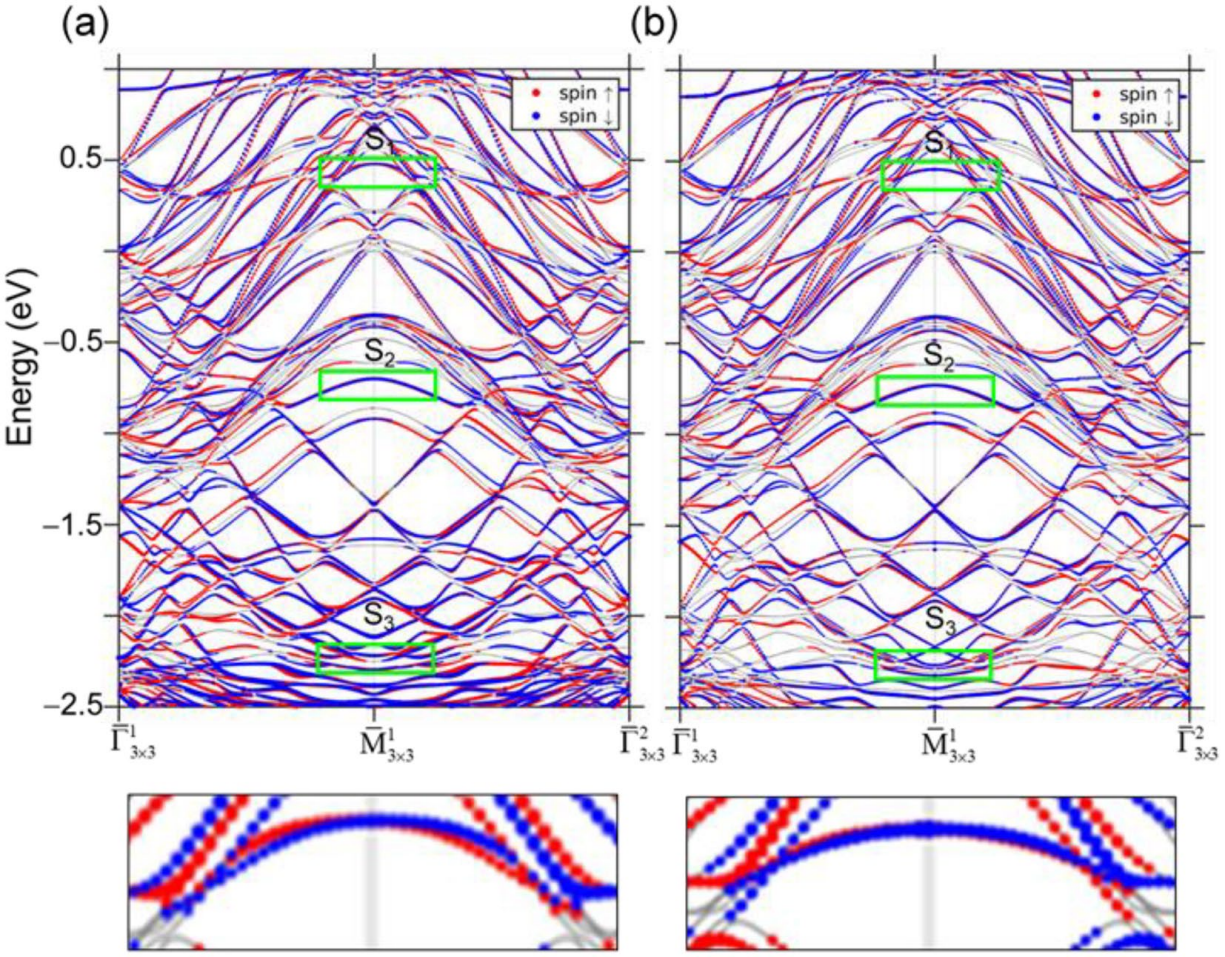
The calculated spin polarization of the (**a**) Cu/Pb(111)-3 × 3 and (**b**) Ag/Pb(111)-3 × 3 energy band structures in the symmetry directions $\overline{\Gamma }_{3 \times 3}^{1} - {\overline{\text{M}}}_{3 \times 3}^{1} - \overline{\Gamma }_{3 \times 3}^{2}$. The insets below show the magnified views of S_1_ bands.

**Figure 6:**
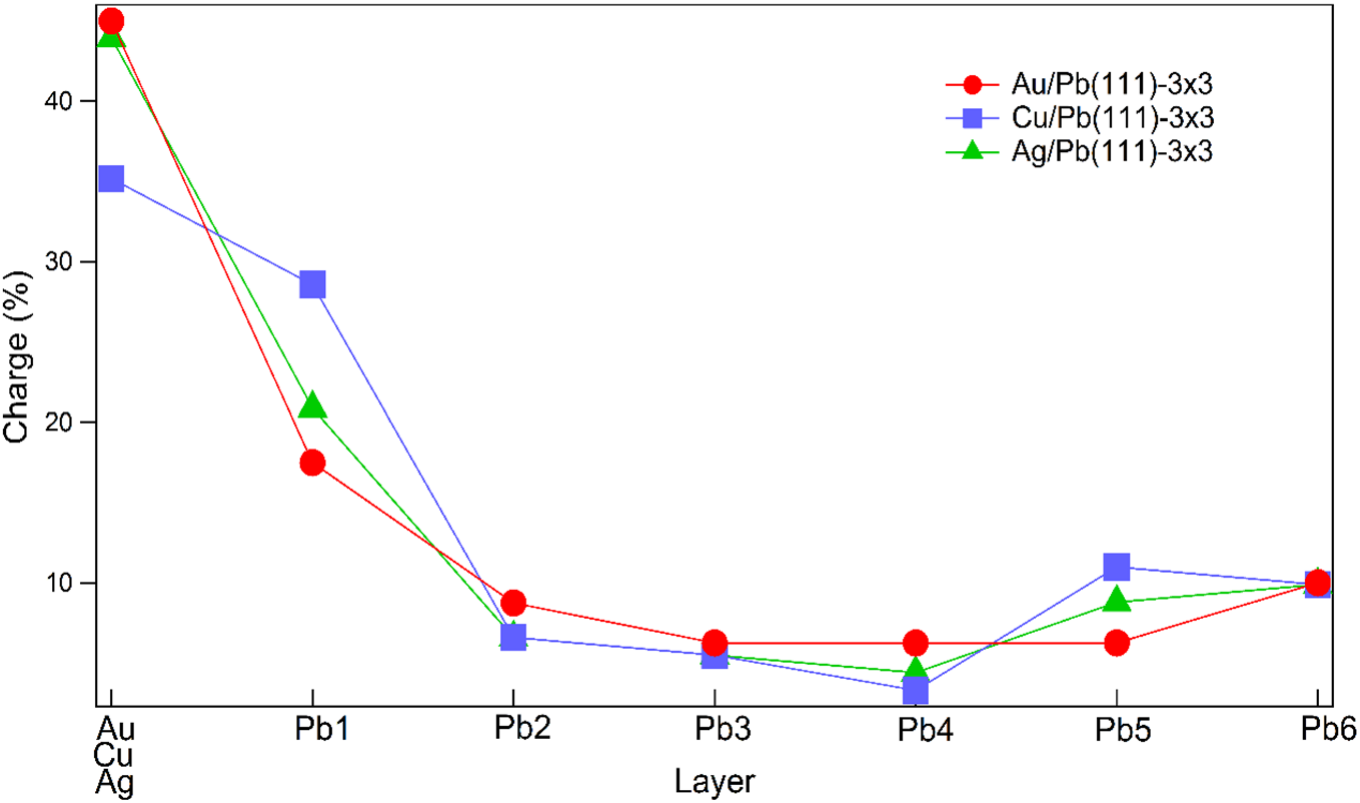
Calculated charge-distribution profile perpendicular to the surface for S_1_ at ${\overline{\text{M}}}_{{{3} \times {3}}}^{{1}}$ (${\overline{\text{M}}}_{{\text{Pb(111)}}}$) in Au/Pb(111)-3 × 3, Cu/Pb(111)-3 × 3 and Ag**/**Pb(111)-3 × 3.

One remaining ambiguity for the Rashba effect is the role of Au that is also a heavy element like Pb (Z_Au_ = 79, Z_Pb_ = 82). Figure 5a,b show the spin-polarization of the calculated subbands for Cu/Pb(111)-3 × 3 and Ag/Pb(111)-3 × 3 in the symmetry direction $\overline{\Gamma }_{3 \times 3}^{1} - {\overline{\text{M}}}_{3 \times 3}^{1} - \overline{\Gamma }_{3 \times 3}^{2}$. Cu and Ag are lighter elements (Z_Cu_ = 29, Z_Ag_ = 47). As indicated by green rectangles, one can spot the corresponding S_1_, S_2_ and S_3_ bands with energies lower than the counterparts of Au/Pb(111)-3 × 3 by ~ 0.3 eV. The S_1_ band is specially magnified to examine the Rashba splitting. Unlike Au/Pb(111)-3 × 3, the S_1_ bands for Cu/Pb(111)-3 × 3 and Ag/Pb(111)-3 × 3 turn to disperse downward with splitting being small for the former and negligible for the latter. It appears that the high *Z* value of Au is important for the observed large Rashba splitting of the S_1_ band in Au/Pb(111)-3 × 3. Nevertheless, it is intriguing that the S_1_ band in Cu/Pb(111)-3 × 3 exhibits a bit more Rashba effect than Ag/Pb(111)-3 × 3 while Cu atom have a much lower *Z* value than Ag. Figure 6 shows the calculated charge distribution of S_1_ state at ${\text{M}}_{{{3} \times {3}}}^{{1}} ({\text{M}}_{{\text{Pb(111)}}} )$ in the Au, Cu, Ag and six Pb layers below, for the three cases. The charge distribution for Cu/Pb(111)-3 × 3 is clearly different, in that the charge percentages at the top Au layer and the first Pb layer are even and hence may assist Rashba effect at the interface. Above all, the actual factors involved in Rashba effect at the interface can be more. For example, pervious investigation of of Au, Cu, and Ag monolayers on W(110)^27,28^ showed the SS of Au monolayer on W(110) had the least Rashba splitting. SOC contribution in opposite directions from Au and W, and the degree of overlayer-substrate hybridization were considered.

## Conclusion

We deposited Au atoms onto Pb(111) surface at 40 K to form a Au/Pb(111)-3 × 3 superstructure. ARPES measurement reveals three distinct SS energy bands (S_1_, S_2_, and S_3_) disperse within and around the rhomboidal bulk energy band gap centered at the surface zone boundary ${\overline{\text{M}}}_{{{3} \times {3}}}^{{1}}$(${\overline{\text{M}}}_{Pb(111)}$). The calculated SS bands based on Au/Pb(111)-3 × 3 on a 6-layer Pb slab match the measured bands well. These three SS bands are composed of mainly Pb *p* orbitals and a few Au *s* and* d* orbitals. S_1_ and S_3_ states of Au/Pb(111)-3 × 3 originate from SRs at the TBBE and BBBE of Pb(111). As revealed from S_1_, the Rashba effect is greatly enhanced (α = 1.0 and 3.54 eVÅ) with respect to that of Au(111) surface and the Pb(111) thin film. Calculation of Rashba splitting for corresponding S_1_ bands in Cu/Pb(111)-3 × 3 and Ag/Pb(111) indicates the important role of Au atoms. Our results show an interesting idea that an irrelevant SR state in a substrate can be promoted by a suitable superstructure or a 2D material on the top, which can further help induce its novel property.

## Experimental procedures and calculation methods

The Pb(111) single crystal substrate was cleaned by repeated cycles of sputtering with a 1.0 keV Ar^+^ ion for an hour followed by annealing at 473 K for 30 min and the surface quality was confirmed by the observations of sharp spots in LEED. During the Au deposition for the formation of Au/Pb(111)-3 × 3 and the subsequent measurements, the temperature of Pb(111) was kept at 40 K using liquid He. When the temperature increases above 40 K, the PbAu alloy starts forming. A water-cooled Knudsen cell was operated at 1523 ± 10 K to deposit Au with a rate of 0.25 Å/min as calibrated from a quartz-crystal thickness monitor. ARPES measurements on Au/Pb(111)-3 × 3 were carried out with a Scienta R4000 energy analyzer using a *p*-polarized light source at 22 eV at the undulator beamline BL21B1 at the National Synchrotron Radiation Research Center (NSRRC) in Taiwan. The energy and angular resolutions were 10 meV and 0.3°.

The ab initio simulations are performed by Vienna Ab initio Simulation Package (VASP)^29,30^ based on density functional theory (DFT). The exchange–correlation functional with Ceperley-Alder (CA) type local-density approximation (LDA)^31^ is utilized in calculations. To simulate the surface-state electronic states, the $1\times 1$ 24-ML Pb(111) slab and $3\times 3$ 6-ML Pb(111) slab with adsorbed Au atoms on top of the Pb slab at the hollow site are considered. The size of the vacuum region used is 15 Å. The gamma-centered Monkhorst–Pack k-meshes of $24\times 24\times 1$ and $12\times 12\times 1$ are used, respectively. The bulk Pb lattice constant of 4.9508 Å and energy cutoff of 300 eV are adopted. The positions of adsorbed Au atoms and top two-layer of Pb are optimized until the residual atomic forces are smaller than 0.03 eV/Å. The unfolding of band structure is carried out by using the BandUP package^32,33^. SOC is applied in the calculation. The calculation of the surface-state electronic states for Cu/Pb(111)-3 × 3 and Ag/Pb(111)-3 × 3 follows the same way.

## Supplementary Information

Supplementary Information.

## Data Availability

The datasets used and/or analyzed during the current study are available from the corresponding author on reasonable request.
