# Author Correction: Formation of surface states on Pb(111) by Au adsorption

**DOI:** 10.1038/s41598-023-30278-8

**Published:** 2023-02-21

**Authors:** Wei‑Chuan Chen, Chin‑Hsuan Chen, Angus Huang, Kaweng Lei, David Mikolas, Ming‑kwan Dai, Je‑Ming Kuo, Dai‑Shien Lin, Cheng‑Maw Cheng, H.‑T. Jeng, S.‑J. Tang

**Affiliations:** 1 Department of Physics, National Tsing Hua University, Hsinchu, 30013 Taiwan; 2 National Synchrotron Radiation Research Center (NSRRC), Hsinchu, 30076 Taiwan; 3 Institute of Physics, Academia Sinica, Taipei, 11529 Taiwan; 4 Physics Division, National Center for Theoretical Sciences, Taipei, 10617 Taiwan

Correction to: *Scientific Reports* 10.1038/s41598-023-28106-0, published online 30 January 2023

The original version of this Article contained errors in the Results and discussion section.

“Wurde et al.^5^ predicted that there two surface resonance (SR) states near TBBE and BBBE at ${\overline{\text{M}}}_{{Pb\left( {111} \right)}}$ [S1 and S2 of Fig. 3 in Ref. 5].”

now reads:

“Wurde et al.^5^ predicted that there are two surface resonance (SR) states near BBBE and TBBE at ${\overline{\text{M}}}_{{Pb\left( {111} \right)}}$ [S1 and S2 of Fig. 3 in Ref. 5].”

Additionally,

“However, their surface weights are not as large as those of S_2_ and S_3_ so the intensity of the extra band is substantially lower than them in Fig. 3c.”

now reads:

“However, their surface weights are not as large as those of S_2_ and S_3_ so the intensity of the extra band is substantially lower than them in Fig. 3d.”

And,

“The blue dashed-dot curves in Fig. 3c, d represent the calculated SR bands of Pb(111), extracted from Fig. 3 of Ref. 5 in two symmetry directions.”

now reads:

“The blue dashed-dot curves in Fig. 3d, f represent the calculated SR bands of Pb(111), extracted from Fig. 3 of Ref. 5 in two symmetry directions.”

Finally,

“Figure 6 shows the calculated charge distribution of S_1_ state at $M_{3 \times 3}^{1} \left( {M_{{Pb\left( {111} \right)}} } \right)$ in the Au and six Pb layers below, for the three cases.”

now reads:

“Figure 6 shows the calculated charge distribution of S_1_ state at $M_{3 \times 3}^{1} \left( {M_{{Pb\left( {111} \right)}} } \right)$ in the Au, Cu, Ag and six Pb layers below, for the three cases.”

The original Article has been corrected.

