# Supplementary material for: Formation of surface states on Pb(111) by Au adsorption
Source: Sci Rep. 2023 Jan 30;13:1689. doi: 10.1038/s41598-023-28106-0 (PMC9886988; doi:10.1038/s41598-023-28106-0)
Supplement: Supplementary file 1 — Supplementary Information. [file 41598_2023_28106_MOESM1_ESM.pdf]

Supplementary Material

Formation of surface states on Pb(111) by Au adsorption

Wei-Chuan Chen[1], Chin-Hsuan Chen[1], Angus Huang[1], Kaweng Lei [1], David Mikolas[1], Ming-kwan Dai[1,] Je-Ming Kuo[1,2], Dai-Shien Lin[1], Cheng-Maw Cheng[2], H -T Jeng[1,3,4 #], S -J Tang[1,2,3*]

1. Department of Physics, National Tsing Hua University, Hsinchu, Taiwan 30013
2. National Synchrotron Radiation Research Center (NSRRC), Hsinchu, Taiwan 30076
3. Institute of Physics, Academia Sinica, Taipei, Taiwan 11529.
4. Physics Division, National Center for Theoretical Sciences, Taipei
, Taiwan 10617.

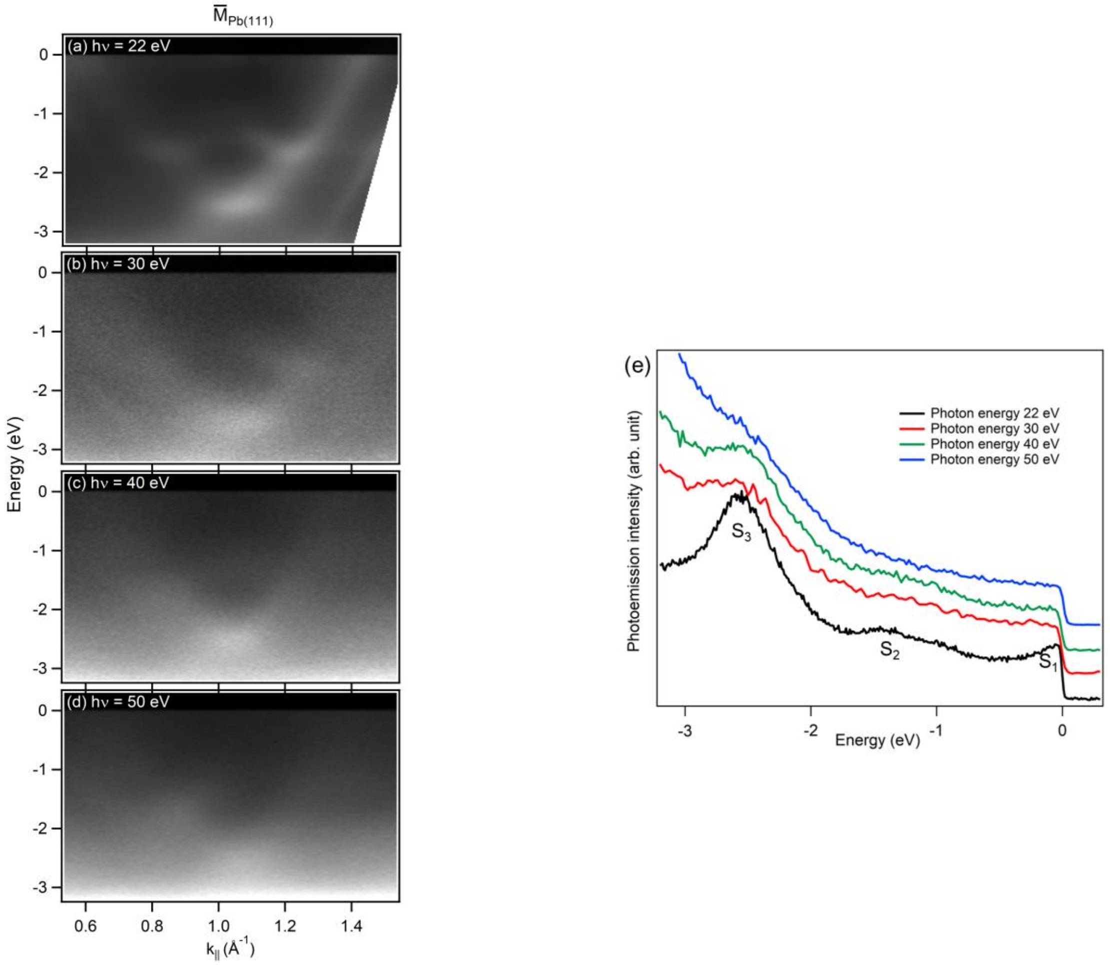

Fig. S1. Photon-energy dependence of S_1, S_2 and S_3 surface states of Au/Pb(111)-3×3. In spite of intensity attenuation, S_1, S_2 and S_3 surface-state bands cantered at $\bar{M}_{Pb(111)}$ don't shift in energy for the photoemission spectra taken at photon energies of (a)22 eV (b) 30 eV(c) 40 eV and (d) 50 eV. (e) The energy distribution curves taken at $k_{||}$= 1.02 Å^{-1}($\bar{M}_{Pb(111)}$) for the four different photon energies. It appears that the photoemission cross section for the S_1, S_2, and S_3 states decrease with increasing photon energy while their energies stay the same.

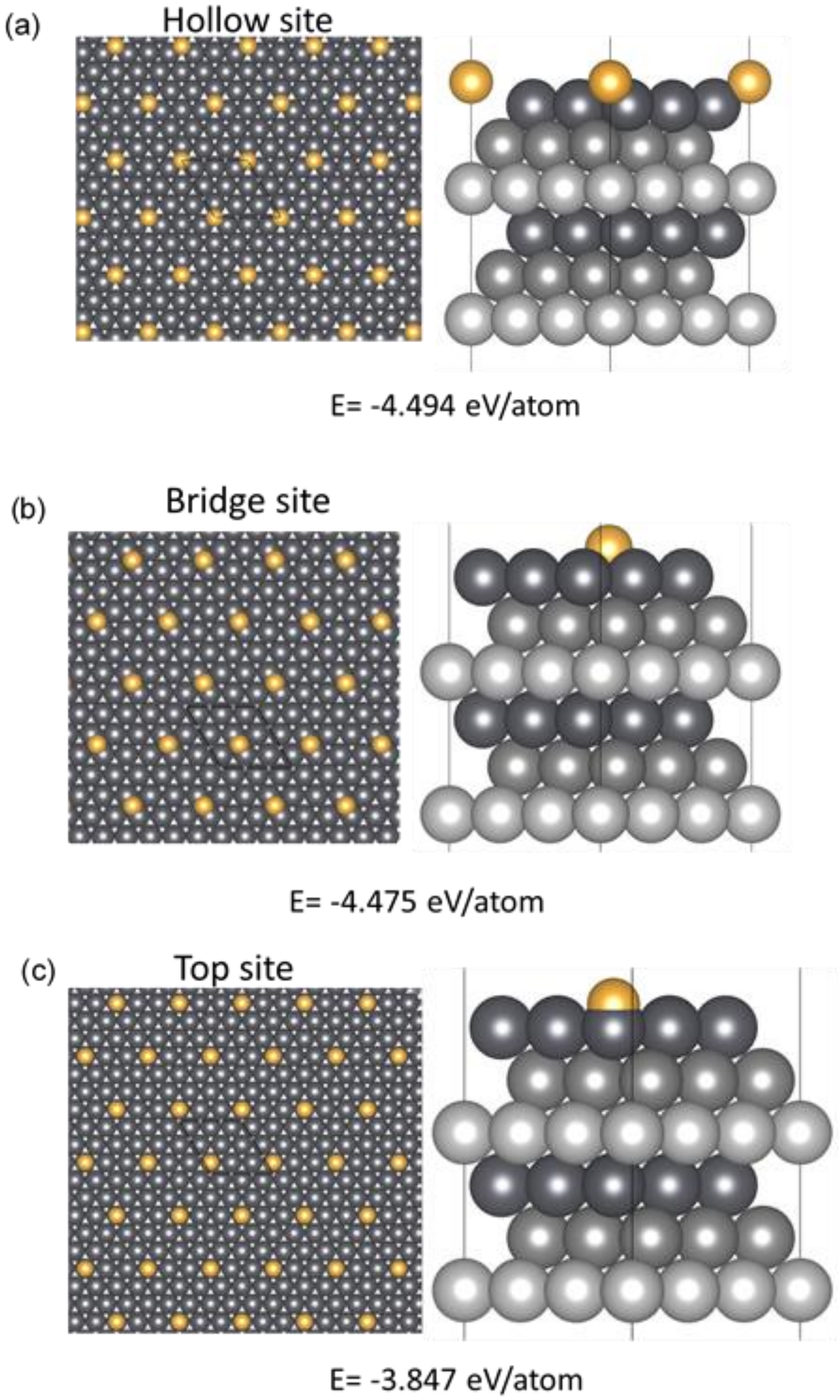

Fig. S2. The three different adsorption configurations of Au/Pb(111)-3×3 for Au atoms at (a) hollow sites, (b) bridge sites, and (c) top sites of Pb(111) lattice with their corresponding calculated energies.

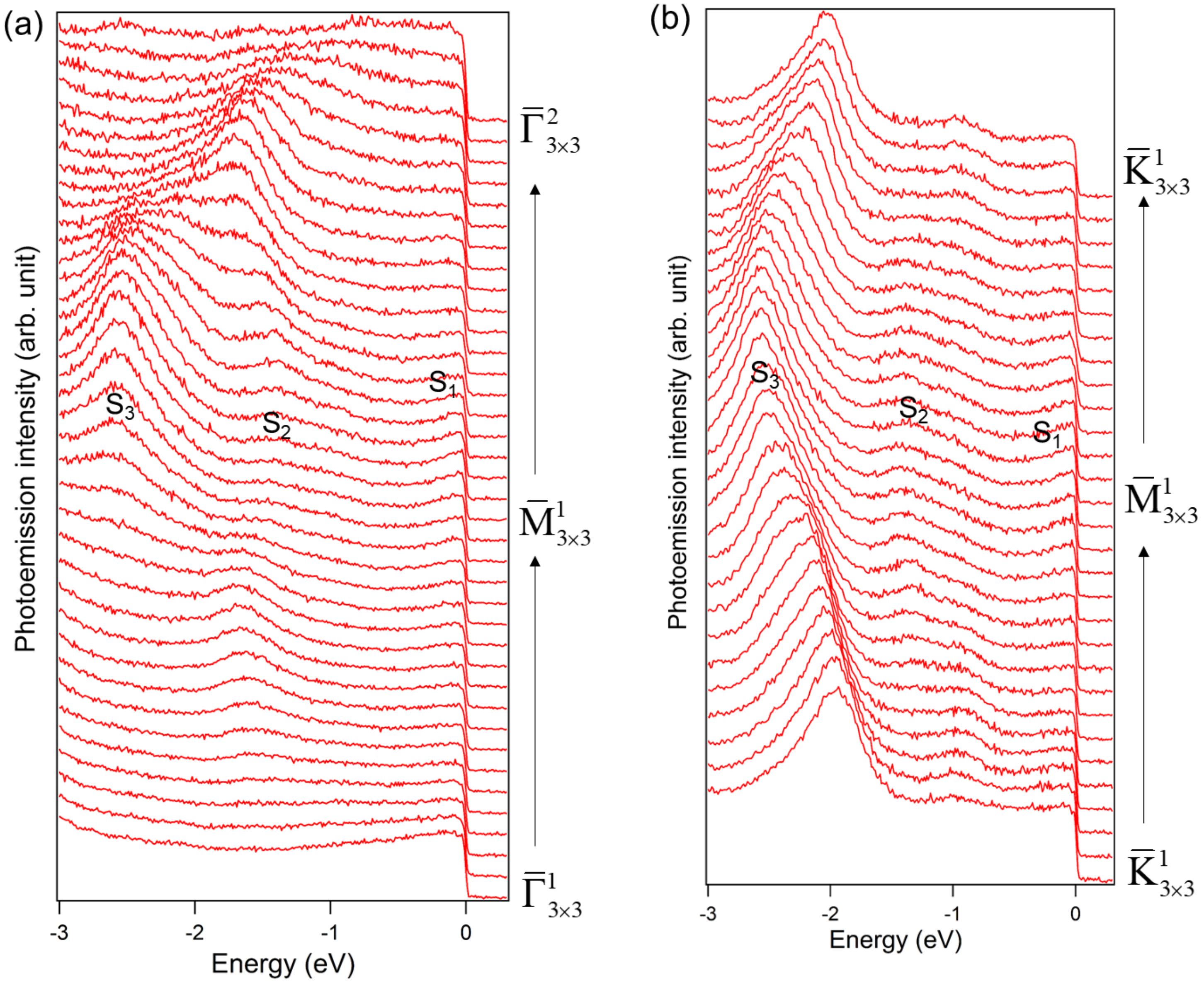

Fig. S3. Photoemission energy distribution curves as a function of *k* momentum in the two symmetry directions (a) $\bar{\Gamma}^1_{3\times3} - \bar{M}^1_{3\times3} - \bar{\Gamma}^2_{3\times3}$ and (b) $\bar{K}^1_{3\times3} - \bar{M}^1_{3\times3} - \bar{K}^1_{3\times3}$ for Au/Pb(111)-3×3.

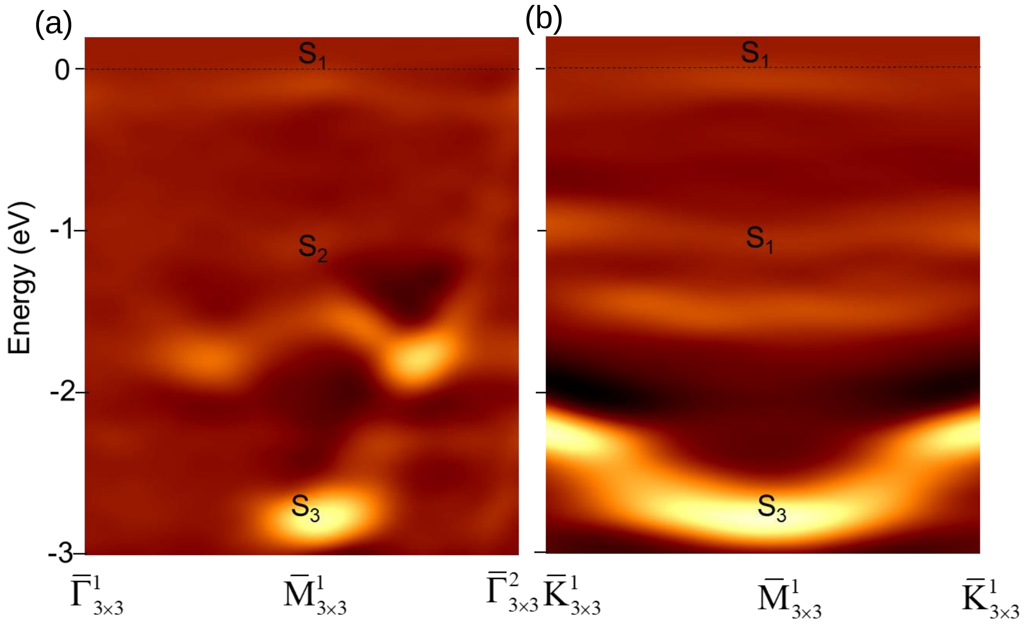

Fig. S4. The measured energy band structures of Au/Pb(111)-3×3 in two symmetry directions (a) $\bar{\Gamma}^1_{3\times3}-\bar{M}^1_{3\times3}-\bar{\Gamma}^2_{3\times3}$ and (b) $\bar{K}^1_{3\times3}-\bar{M}^1_{3\times3}-\bar{K}^1_{3\times3}$ after the 2nd-derivative image processing.

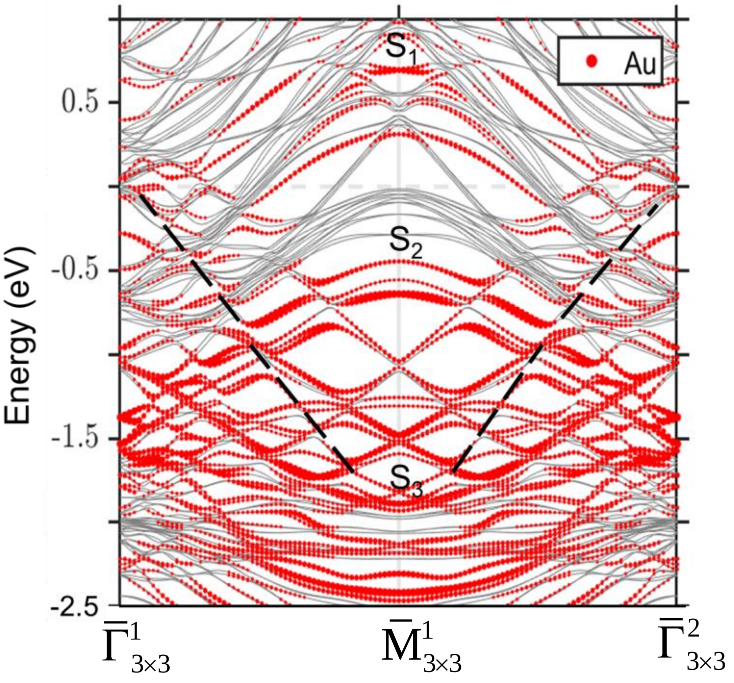

Fig. S5. The calculated bands of Au/Pb(111)-3x3 with the red color indicating the weight on the top Au atoms. The dashed lines indicate the extra bands observed in the measurement.

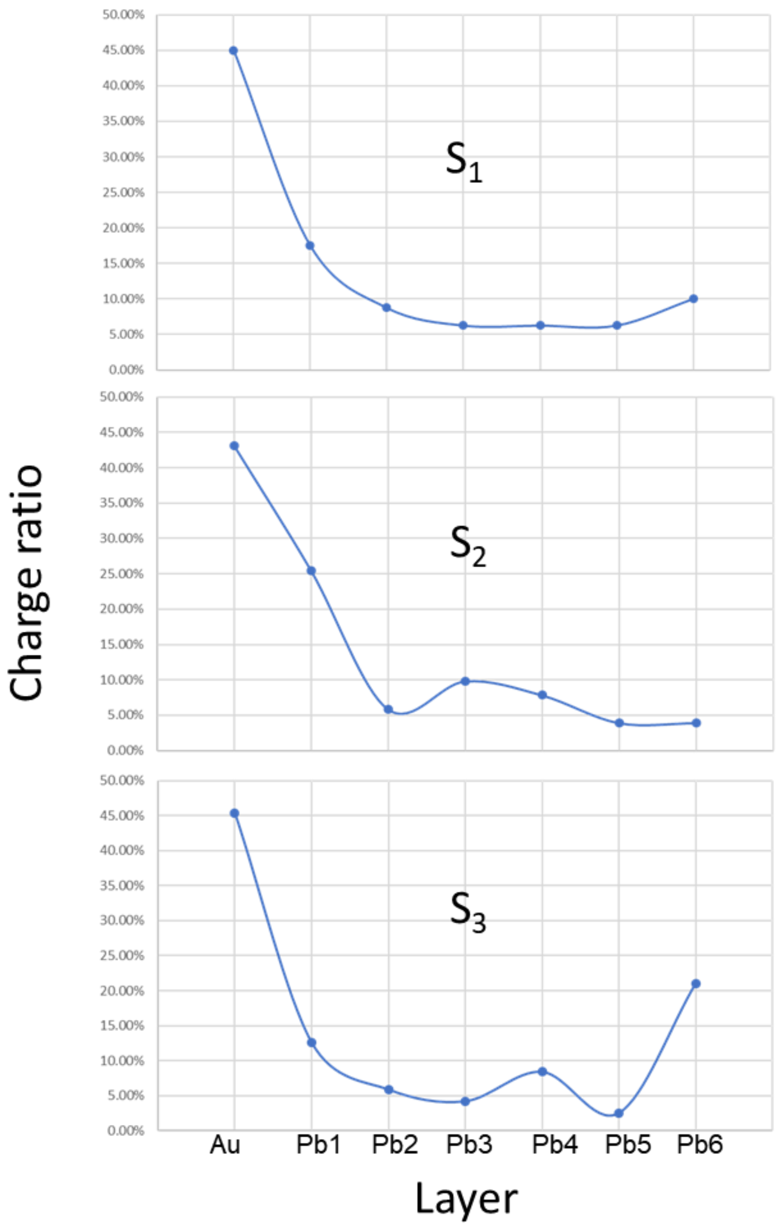

Fig. S6. Charge-distribution profile perpendicular to the surface for S_1, S_2, and S_3 states at $\overline{M}^{1}_{3\times 3}$ ($\overline{M}_{Pb(111)}$).
